# Genomic alterations in abnormal neutrophils isolated from adult patients with systemic lupus erythematosus

**DOI:** 10.1186/ar4681

**Published:** 2014-08-08

**Authors:** Namrata Singh, Pamela Traisak, Kayla A Martin, Mariana J Kaplan, Philip L Cohen, Michael F Denny

**Affiliations:** Section of Rheumatology, Temple University, 3322 North Broad Street, Philadelphia, PA 19140 USA; Department of Microbiology and Immunology, Temple University, 3500 North Broad Street, Philadelphia, PA 19140 USA; Systemic Autoimmunity Branch, Intramural Research Program, NIAMS/NIH, 10 Center Drive, Bethesda, MD 20892 USA; Temple Autoimmunity Center, Temple University, 3500 North Broad Street, Philadelphia, PA 19140 USA

## Abstract

**Introduction:**

Patients with systemic lupus erythematosus (SLE) have an abnormal population of neutrophils, called low-density granulocytes (LDGs), that express the surface markers of mature neutrophils, yet their nuclear morphology resembles an immature cell. Because a similar discrepancy in maturation status is observed in myelodysplasias, and disruption of neutrophil development is frequently associated with genomic alterations, genomic DNA isolated from autologous pairs of LDGs and normal-density neutrophils was compared for genomic changes.

**Methods:**

Alterations in copy number and losses of heterozygosity (LOH) were detected by cytogenetic microarray analysis. Microsatellite instability (MSI) was detected by capillary gel electrophoresis of fluorescently labeled PCR products.

**Results:**

Control neutrophils and normal-density SLE neutrophils had similar levels of copy number variations, while the autologous SLE LDGs had an over twofold greater number of copy number alterations per genome. The additional copy number alterations found in LDGs were prevalent in six of the thirteen SLE patients, and occurred preferentially on chromosome 19, 17, 8, and X. These same SLE patients also displayed an increase in LOH. Several SLE patients had a common LOH on chromosome 5q that includes several cytokine genes and a DNA repair enzyme. In addition, three SLE patients displayed MSI. Two patients displayed MSI in greater than one marker, and one patient had MSI and increased copy number alterations. No correlations between genomic instability and immunosuppressive drugs, disease activity or disease manifestations were apparent.

**Conclusions:**

The increased level of copy number alterations and LOH in the LDG samples relative to autologous normal-density SLE neutrophils suggests somatic alterations that are consistent with DNA strand break repair, while MSI suggests a replication error-prone status. Thus, the LDGs isolated have elevated levels of somatic alterations that are consistent with genetic damage or genomic instability. This suggests that the LDGs in adult SLE patients are derived from cell progenitors that are distinct from the autologous normal-density neutrophils, and may reflect a role for genomic instability in the disease.

**Electronic supplementary material:**

The online version of this article (doi:10.1186/ar4681) contains supplementary material, which is available to authorized users.

## Introduction

Systemic lupus erythematosus (SLE) is an autoimmune disease of complex etiology. Intense and ongoing research efforts into the genetics of SLE have greatly advanced our understanding of the susceptibility to and development of the disease [1, 2]. More recently, research emphasis has shifted toward the identification and characterization of causative genetic alterations that convey the associated risk linkages in human SLE [3], as well as mouse models of disease [4]. As research into the genetics of SLE continues, and the resolution of analysis becomes more refined, it is becoming increasingly apparent that many of the identified susceptibility intervals provide a rather limited contribution to disease incidence when considered individually [5]. As such, models of SLE development generally propose that in the majority of cases, it is the inheritance of a combination of multiple susceptibility intervals that actually drives the development of disease in any one individual [6, 7]. The profile of these inherited genetic intervals has also been proposed to influence the manifestations of the disease, such as time to onset, disease progression and target organ involvement [8]. Despite all of these advances that have established the role of genetics in SLE, inheritance alone rarely accounts for the incidence of SLE in an individual, suggesting that there are additional influences that contribute to the disease.

Because SLE is typically characterized by the progressive development of autoantibodies that recognize components of the cell nucleus [9], it is frequently considered to result from a disruption in the regulation of the adaptive immune system [10]. However, recent evidence supports additional contributions from components of the innate immune system [11]. Research toward defining the importance of additional cell lineages in the development of SLE is ongoing, and alterations in granulocyte function have been identified in both pediatric and adult SLE [12–15]. In this regard, an intriguing population of abnormal granulocytes has been identified and isolated from SLE patients [12–14, 16, 17]. These low-density granulocytes (LDGs) contribute the granulocyte signature observed in gene expression arrays from the mononuclear cell fraction of pediatric lupus patients [17]. In adults, LDGs mediate enhanced proinflammatory and cytotoxic responses compared to those of autologous normal-density neutrophils [12, 13]. These LDGs readily induce endothelial cell death and spontaneously form neutrophil extracellular traps (NETs) [12, 13]. Despite these advances in our understanding of the function of LDGs, their developmental origins remain undefined. One model proposes that LDGs arise as a consequence of *in situ* activation of normal neutrophils [15]. However, direct gene expression array analysis and bioinfomatic pathway comparisons between autologous pairs of LDGs and normal-density neutrophils isolated from SLE patients were not entirely consistent with an *in situ* activation model [13]. The expression of genes associated with mature neutrophils did not differ between the LDGs and autologous normal-density neutrophils, nonetheless genes related to azurophilic granules, as well as cytoskeleton and adhesion molecules, were altered [13]. Thus, mechanisms other than *in situ* activation may contribute to the development of LDGs in SLE patients.

One such alternate mechanism for the production of LDGs would be a disruption in granulocyte development. While the initial characterizations of LDGs suggested that they were immature granulocytes, based upon their lobulated or ovoid nuclei [14, 17], further characterization of the surface molecule expression on LDGs revealed a profile that is more consistent with a fully mature developmental state [12, 13]. This apparent inconsistency in maturation phenotype has also been described in patients with myelodysplasias [18]. While the genetics of SLE is a topic of intense interest, the potential contributions of genetic instability and somatic mutations in SLE have received far less attention [19], in large part due to the difficulties associated with clearly identifying altered cell lineages and applying techniques that are suitable for analysis [20–22]. Since we have previously developed techniques to isolate highly enriched autologous pairs of normal-density neutrophils and LDGs from individual SLE patients [12, 13], it was now possible to apply genomic techniques currently utilized in cancer research to examine SLE LDGs for evidence of genomic instability [23]. We hypothesized that the LDGs arise from a process that is similar to myelodysplasia, and therefore genomic alterations should exist between the autologous pairs of LDGs and normal-density neutrophils isolated from individual SLE patients. We report herein that there is clear evidence for genetic instability within the LDG lineage in SLE patients, with multiple types of genomic abnormalities detected in some SLE patients.

## Methods

### Recruitment of SLE patients and healthy controls

The Institutional Review Boards at Temple University and the University of Michigan approved this study. Subjects gave informed consent in accordance with the Declaration of Helsinki. Lupus patients fulfilled the revised American College of Rheumatology criteria for SLE and enrollment was open to all patients examined at the outpatient rheumatology clinics at Temple University and the University of Michigan [24]. Disease activity was assessed by the SLE disease activity index (SLEDAI) [25]. Female healthy controls were recruited by advertisement. Demographic and clinical information for the lupus patients enrolled in the study (including medications) were extracted from patient charts.

### Isolation of LDGs and neutrophils

LDGs and autologous neutrophils were isolated from the blood of SLE patients as described previously [12, 13]. Briefly, venous blood (approximately 60 ml) was collected in heparinized tubes and separated by discontinuous density gradient centrifugation using Ficoll-Hypaque. LDGs were isolated from the PBMC layer by negative selection of lymphocytes and monocytes using a panel of biotinylated antibodies recognizing CD3, CD7, CD19, CD56, CD79b, CD86, MHC class II, and erythrophorin (Ancell, Bayport, MN, USA). The labeled cells were depleted using paramagnetic beads coupled with an anti-biotin antibody and a magnetic column (Miltenyi, Bergisch Gladbach, Germany). Normal density neutrophils were recovered from the corresponding erythrocyte fraction of the Ficoll-Hypaque gradient by dextran sedimentation of erythrocytes and lysis of residual red blood cells [26]. The resultant cell purity of LDGs and autologous neutrophils exceeded 90% as assessed by flow cytometric analysis using the monocyte marker CD14 and the neutrophil marker CD15 [12].

### Isolation of genomic DNA

Purified LDGs and neutrophils (5 to 20 X 10^6^) were incubated at 65°C overnight in 0.4 ml of cell lysis buffer containing proteinase K (100 mMTris, pH 8.0, 0.2% sodium dodecyl sulfate, 5 mM EDTA, 200 mMNaCl, 0.4 mg proteinase K/mL (Sigma-Aldrich, St Louis, MO, USA)) [27]. After cooling, denatured proteins were removed by phenol-chloroform extraction, and the aqueous phase was collected. Total nucleic acids were precipitated by addition of an equal volume of isopropanol, and recovered by centrifugation. The pellet was washed once in 70% ethanol, air-dried, dissolved in water, and treated with DNAse-free RNAse (Promega Madison, WI, USA) for 30 min at 37°C. Purified genomic (g)DNA was precipitated by addition of three volumes of ethanol, incubated at −20°C overnight, recovered by centrifugation, and the pellet stored in 70% ethanol at −20°C. The purity and integrity of the gDNA was assessed by agarose gel electrophoresis. All samples of gDNA were free of residual RNA and displayed a single band of greater the 24 kb (see Additional file 1).

### Cytogenetic microarray analysis

Cytogenetic microarray analysis was performed by the Cytogenetics and Chromosomal Microarray core at the Fox Chase Cancer Center, using the Affymetrix 2.7 M Cytogenetics array chip, and genomic alterations were identified using Affymetrix Chromosome Analysis Suite software (Version 2.1) (Affymetrix, Santa Clara, CA, USA). This microarray chip evaluates genomic segments and single nucleotide polymorphism (SNP) markers, thus it is capable of simultaneously identifying both copy number alterations, such as duplications and deletions, as well as copy number-neutral losses of heterozygosity (LOH) [28–31]. Copy number alterations that were at least 9 kb in length and detected by a minimum of 10 consecutive markers with 80% confidence were included. Copy number alterations that spanned the centromere were excluded. Likewise, since only female subjects were used in this study, any interval that was localized to the Y chromosome was eliminated. Previously established and novel genomic variations were included in the analysis in order to distinguish inherited copy number variants from somatic alterations in the SLE patients. Each pair of SLE LDG and neutrophil samples was processed and analyzed together to minimize variability. The SNP markers on the microarray chip were also used to identify copy number-neutral LOH. Intervals greater than 2 Mb in length and detected with a minimum of 80% confidence were included in the analysis. Regions of LOH were compared between each SLE patient’s LDG and neutrophil samples to identify constitutional LOH from somatic mutations.

### PCR analysis of JAK2V617F mutation and Flt3 alterations

Somatic mutations resulting in the conversion of wild-type JAK2 to a dominant activated form (JAK2V617F) were assessed in the pairs of LDGs and normal-density neutrophils by tetra-primer amplification refractory mutational screening using established primers and PCR conditions [32, 33]. The JAK2V617F positive cell line HEL served as a control. Flt3 mutation within the kinase domain activation loop was also tested by PCR [34]. The introduction of an aspartate at amino acid position 835 was examined by the loss of an EcoRV restriction site encoded in Flt3 [35]. Flt3 internal tandem duplications in the juxtamembrane region were evaluated by an alteration in the size of the PCR amplicon in controls and SLE samples [35].

### Microsatellite instability (MSI) assays

A total of six microsatellites were analyzed using fluorescence-labeled PCR primers and capillary gel electrophoresis. The primer sequences and PCR protocol for five quasimonomorphic microsatellites (NR21, NR22, NR24, BAT25 and BAT26) and one polymorphic microsatellite (BAT40) have been described previously [36–39]. PCR products for both the LDGs and autologous neutrophils were compared. MSI was determined based upon differences in the main PCR product peak identified for each amplicon from the autologous pairs of LDGs and normal-density neutrophils for each SLE patient. A sample of genomic DNA isolated from the replication error-prone cell line Jurkat was amplified and analyzed in parallel with each set of patient samples to confirm the reproducibility of the MSI assay [40].

### Statistical analysis

Because the distribution of copy number alterations is noncontinuous, only nonparametric analysis could be applied. Pairwise comparisons between autologous sets of LDGs and normal-density neutrophils were performed using Wilcoxon signed-rank test, with a one-tailed *P* value <0.05 considered statistically significant.

## Results

### Copy number alterations are present in LDGs isolated from human SLE patients

**Figure 1:**
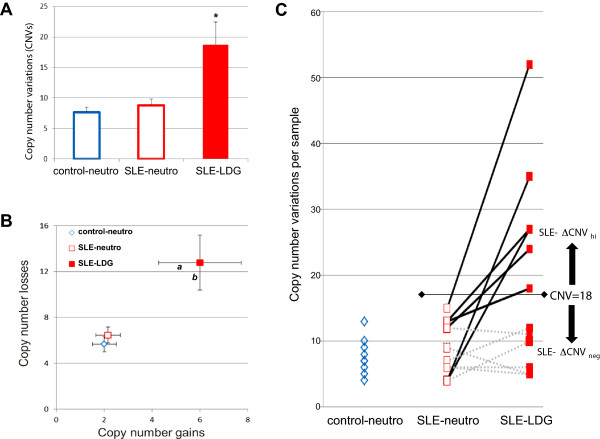
**LDGs isolated from SLE patients have greater levels of copy number alterations relative to control neutrophils and autologous neutrophils. (A)** Genomic DNA from nine healthy female donors and thirteen SLE patients was analyzed by cytogenetic microarray analysis. Values are mean ± SEM. *Distribution differs significantly from autologous normal-density SLE neutrophils, Wilcoxon signed-rank test, one-tailed, *P* <0.01. **(B)** The incidence of genomic duplications and deletions was similar in the neutrophil samples isolated from the healthy controls and SLE patients, whereas the autologous LDGs had a significant increases in the number of deletions (a) Wilcoxon signed-rank test, one-tailed, *P* <0.01) and duplications (b) Wilcoxon signed-rank test, one-tailed, *P* <0.01). Values are mean ± SEM. **(C)** Copy number variations for each healthy control (open blue diamonds, *n* = 9), and autologous pairs of SLE neutrophils (open red squares) and LDGs (filled red squares) are indicated. LDG samples with 18 or more copy number variations were considered ∆CNV_hi_. Autologous sample pairs for each SLE patient are indicated by a solid black line for ∆CNV_hi_, and a dashed gray line for the ∆CNV_neg_. CNV, copy number variation; LDG, low-density granulocyte; SEM, standard error of the mean; SLE, systemic lupus erythematosus.

To determine whether the LDGs isolated from human SLE patients have evidence of genomic instability, we applied diagnostic techniques commonly utilized in cancer research. DNA from thirteen female SLE patients was analyzed for genomic alterations, compared to DNA isolated from the neutrophils of nine healthy female donors. Cytogenetic microarray analysis was used to identify copy number alterations (duplications and deletions), as well as copy number-neutral LOH, in genomic DNA isolated from autologous pairs of LDGs and normal-density neutrophils. Autologous pairwise comparisons of the genomic profile of each SLE patient’s LDGs to their normal-density neutrophils distinguish heritable copy number variations from *bona fide* somatic mutations. A total of 69 copy number variations were identified in healthy controls (*n* = 9), and 114 and 244 alterations, respectively, were detected in normal-density neutrophils and LDG fractions isolated from SLE patients (*n* = 13). This corresponded to an average of 7.67 chromosomal copy number variations per diploid genome in control neutrophils, similar to the average present in the SLE neutrophils value of 8.77 variations. In sharp contrast, the autologous SLE LDGs had an average of 18.77 variations per genome (Figure 1A). This increased frequency of copy number alterations in the LDGs was comprised of an elevation in the number of deletions and duplications (Figure 1B). Thus, the LDGs have copy number alterations that are not present in autologous normal-density neutrophils, consistent with genomic instability.

Closer inspection of the individual SLE sample pairs revealed two distinct profiles for the copy number alterations in the LDGs (Figure 1C). The majority of the alterations were found in a subset of SLE patients. Because healthy donors had an average of number of copy number alterations of 7.67 ± 2.58 (mean ± standard deviation (SD)), we considered a SLE LDG sample as ‘change in copy number variation high’ (∆CNV_hi_) if it possessed a total number of copy number alterations that exceeded four standard deviations above the mean number of sites present in control neutrophils. Thus, any SLE LDG sample that had 18 or more copy number alterations was considered ∆CNV_hi_. Using this criterion, seven of the thirteen SLE samples had levels of copy number variations in their LDGs that were equivalent to autologous normal-density neutrophils and healthy controls (see Additional file 2). The remaining six SLE patient samples had levels of copy number alterations in their LDG fraction that exceeded the benchmark of eighteen (range 18 to 52). It was noteworthy that the level of copy number alterations detected in the normal-density neutrophils isolated from the ∆CNV_hi_ subset of SLE patients was similar to healthy donors (Figure 1C). Therefore, the changes in copy number seen in lupus were restricted to the LDGs, with the corresponding normal-density neutrophils isolated from these SLE patients possessing similar levels of copy number variations as healthy controls, or samples from the ∆CNV_neg_ SLE patients (Figure 1C). Therefore, there was a marked increased frequency of alterations in copy number alterations in six of the thirty SLE samples, consistent with genomic instability.

### Copy number alterations in LDGs are localized to chromosomes and genomic intervals

**Figure 2:**
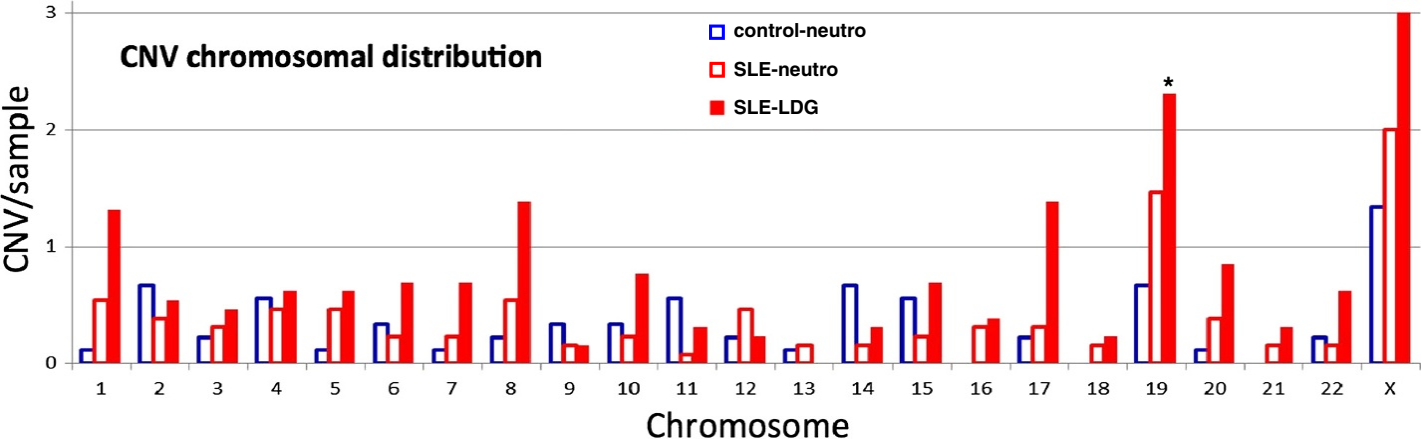
**Selective distribution of acquired copy number variations in LDGs to specific chromosomes.** The frequency of copy number variations in healthy controls (open blue bar), SLE neutrophils (open red bar) and autologous LDGs (filled red bar) are displayed. *Significant increase in copy number alterations located on chromosome 19 were detected in LDGs relative to control neutrophils (Kruskal-Wallis test with Mann-Whitney *post hoc* analysis, one-tailed, *P* <0.01). Trends for increases levels of CNVs in the LDGs were also suggested for chromosomes 8, 17, and X. CNV, copy number variation; LDG, low-density granulocyte; SLE, systemic lupus erythematosus.

The distribution of the alterations in copy number was assessed to determine if they were evenly, or randomly, distributed across the entire genome or preferentially localized to particular chromosomes or genomic regions. The chromosomal alterations in the LDGs were most prominent on relatively few chromosomes, including chromosomes 8, 17, 19 and X (Figure 2), consistent with a nonrandom distribution profile. Because the size of each individual chromosome varies, the frequency for each chromosomal alteration was normalized for relative size. For example, while chromosome 1 had an apparent increase in the number of genomic alterations in LDGs, it was approximately equivalent to the predicted value based upon random distribution after normalization for the large size of chromosome 1. When adjusted for chromosomal size, chromosome 19 had the greatest relative increase above the predicted incidence with over five-fold more variations per sample than predicted by the random normalized distribution (Figure 2). The X chromosome harbored the highest average incidence of variations with three copy number alterations per sample, but this corresponded to a relative increase in X chromosome alterations of slightly greater than three-fold above the predicted value. The frequency of copy number variations on the remaining chromosomes was similar among the healthy control neutrophils, and SLE normal-density neutrophils and LDGs. Thus, the LDGs displayed a marked increase in somatic alterations on specific chromosomes when compared to either autologous normal-density neutrophils or healthy controls.

**Figure 3:**
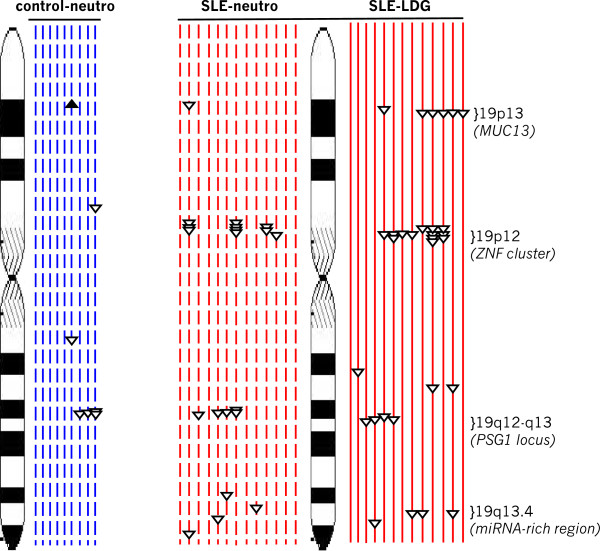
**The copy number alterations on chromosome 19 are localized to particular intervals.** Chromosome 19 is shown in detail, and the relative locations of individual duplications (upward filled arrowheads) and deletions (downward open arrowheads) are indicated for each healthy control sample (dashed blue line adjacent to ideogram), SLE neutrophil (dashed red line) and autologous LDG sample (solid red line). CNVs in the LDGs were predominantly within regions in 19p13 encoding the *MUC16* gene, intergenic intervals in 19p12 within a *ZNF* gene cluster, 19q12-q13 containing several Pregnancy-specific glycoproteins, and 19q13.4 amid an miRNA cluster. CNV, copy number variation; LDG, low-density granulocyte; miRNA, microRNA; SLE, systemic lupus erythematosus.

The genomic alterations were restricted to relatively few genetic intervals on specific chromosomes. Of note, the somatic alterations identified on chromosome 19 were deletions that clustered within four intervals (Figure 3). None of these affected regions on chromosome 19 have been recorded in the Database of Genomic Variations, maintained by the University of Toronto [41]. Three of the LDG-specific chromosome 19 deletions included a loss of exon 3 of the MUC16 gene. MUC16 encodes the CA125 antigen, a diagnostic marker in ovarian cancer screening and recently reported to be altered in SLE patients, leading to the inclusion of SLE as a confounder in interpretation of CA125 screening [42]. A second interval of chromosome 19 deletions in LDGs was concentrated within a cluster of zinc-finger transcription factors within 19p12 [43]. The number of 19p12 deletions ranged from one to four, and seven LDG samples had deletions in the intergenic regions between individual zinc-finger transcription factions. A deletion between ZNF99 and ZNF492 was identified in six of the thirteen LDG samples, but not in any of the nine controls. Two additional sites of common LDG deletions were found on 19q. One site was within the Pregnancy-specific glycoprotein 1 gene cluster, and another was within an miRNA-dense region [44]. Another noteworthy set of alterations was identified on chromosome 17, in which LDGs from two patients possessed a somatic duplication of the retinoic-acid receptor alpha (not shown).

### Copy number-neutral losses of heterozygosity (LOH) are also present in LDGs isolated from human SLE patients

**Figure 4:**
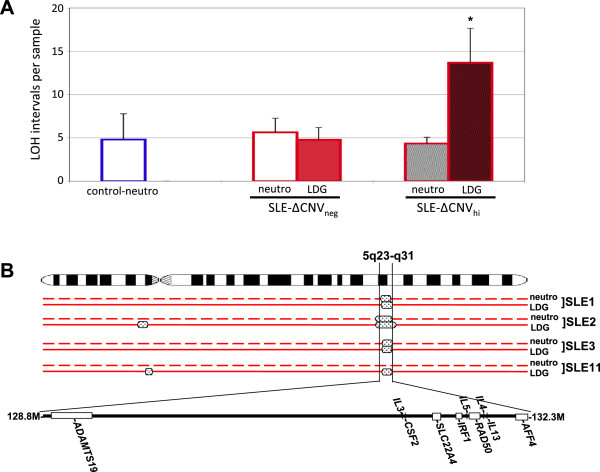
**Losses of heterozygosity (LOH) in LDGs from SLE patients. (A)** LOH are prevalent in the ∆CNV_hi_ SLE patients. The mean ± SEM of LOH >2 Mb for healthy controls (open blue bar, *n* = 9), SLE neutrophils (red open bar) and SLE LDGs (red filled bar). ∆CNV_neg_ (*n* = 7) and ∆CNV_hi_ (*n* = 6) SLE patients are indicated by unhatched and black-hatched bars, respectively. *Differs significantly from autologous normal-density SLE neutrophils, Wilcoxon signed-rank test, one-tailed, *P* <0.01. **(B)** A LOH at 5q23-q31 is observed in four SLE patient samples. The autologous pairs of neutrophils and LDGs are indicated by the dashed red line and solid red line, respectively. The affected 3.5 Mb interval is expanded to show the relative positions of genes indicated in the text, additional genes within the interval are not shown for the purposes of clarity. SLE patient 11 has a loss of heterozygosity that is restricted to the LDG sample. Refer to Additional file 5 information regarding SLE patients. Genomic positions based upon Human Genome Assembly Build 36.3. CNV, copy number variation; LDG, low-density granulocyte; SEM, standard error of the mean; SLE, systemic lupus erythematosus.

The Affymetrix 2.7 M cytogenetic microarray chip also detects copy number-neutral LOH. While the sensitivity of the chip for the detection of LOH is lower than that for copy number alterations, it is sufficient to identify large-scale LOH (>2 Mb). The overall level of heterozygosity at the genomic, or individual chromosomal level, did not differ among control neutrophils, SLE normal-density neutrophils and autologous LDGs (not shown), suggesting that uniparental disomy resulting from break-induced replication was not present in LDGs. Rather, the previously identified ∆CNV_hi_ SLE patients also had a higher incidence of LOH, again consistent with accumulated somatic damage (Figure 4A). A distinct region of LOH in 5q23.3-5q31.1 was observed in four of thirteen SLE patients, but not in any of the nine healthy controls (Figure 4B). The affected 5q LOH interval harbors several cytokine genes (including IL-3, IL-4, IL-5, IL-13, and CSF2), a gene associated with arthritis susceptibility (SLC22A4), a DNA repair gene (RAD50), and a gene that promotes Type 1 interferon responses (IRF1) [45–50]. Of the four SLE samples possessing a region of 5q LOH, two were constitutive LOH from patients that did not display an increase in copy number alterations, one had increased copy number alterations, and one SLE sample had a somatic 5q LOH restricted to the LDGs that was not present in the autologous normal-density neutrophils. Thus, using cytogenetic microarray analysis, we identified SLE patients with a propensity for genomic instability that included copy number alterations and LOH, consistent with repair of DNA strand breaks.

### Activating mutations in JAK2 and Flt3 are not observed in LDGs

Due to the genomic alterations within the LDGs isolated from SLE patients, it is possible these cells also possess specific genomic alterations that affect neutrophil development. Myeloproliferative disorders are associated with a somatic mutation in the JAK2 kinase [51]. JAK2 V617F displays constitutive activation that promotes the expansion of the erythroid and myeloid compartments [52]. The LDGs and autologous normal-density neutrophils isolated from SLE patients were examined for JAK2 V617F mutation by tetra-primer amplification refractory mutation system (ARMS) assay (see Additional file 3). None of the samples displayed the JAK2 mutation, indicating that the LDGs did not possess the activated form of the JAK2 kinase. In addition, mutations in the Flt3 receptor kinase domain were examined by PCR [35]. An activating point mutation at D835 within the kinase domain and internal tandem repeats of the juxtamembrane region were assayed, but again none of the sample pairs displayed either of these alterations (see Additional file 4). Thus, while a subset of the LDGs has an increased frequency of somatic errors including duplications, deletions, and LOH, specific mutations in either the JAK2 kinase or Flt3 kinase that have been associated with myeloproliferative disorders or acute myeloid leukemia were not detected.

### Microsatellite instability (MSI) is present in LDGs isolated from human SLE patients

**Figure 5:**
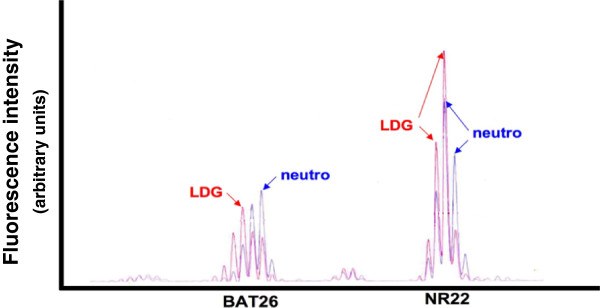
**Microsatellite instability (MSI) in SLE LDGs.** Representative chromatographic traces for capillary gel electrophoresis analysis of duplex PCR reactions for the quasimonomorphic BAT25 and NR22 microsatellites. The size of the BAT25 amplicon differs between an autologous pair of SLE neutrophils (red line) and LDGs (blue line). The NR22 product was scored as stable in this SLE patient since the primary peak in the LDGs is the same size as the autologous normal-density neutrophils. LDG, low-density granulocyte; SLE, systemic lupus erythematosus.

While the cytogenetic microarray assays are quite sensitive for the detection of copy number alterations and LOH, they are not designed to identify all types of genomic alterations. MSI is frequently indicative of a defect in DNA mismatch repair during replication, either through inheritance of nonfunctional DNA repair genes or through somatic inactivation of repair genes within the cancer cell progenitor [36]. Because the LDGs demonstrated genomic alterations consistent with increased incidence of DNA strand breaks, a set of six microsatellite markers was analyzed for evidence of instability as an indicator of replication error. This panel included five quasimonomorphic mononucleotide microsatellites used in the diagnosis of DNA replication error-prone tumors (BAT25, BAT26, NR21, NR22, NR24), as well as a polymorphic microsatellite (BAT40). The microsatellites were amplified using fluorescently labeled PCR primers, and the sizes of the amplicons were evaluated by capillary gel electrophoresis. MSI in the SLE patients was evaluated by comparing the size of the PCR product obtained from the LDG sample to that of the autologous normal-density neutrophil. Three SLE patients displayed clear size shifts in the main product peak. In total, six microsatellites displayed instability, with one patient having three unstable microsatellites, another with two, and a third with a single unstable microsatellite (Figure 5). The SLE patient with three unstable microsatellites was also ∆CNV_hi_ and possessed an LDG-restricted 5q LOH. The patient with one unstable microsatellite marker also had an accompanying 5q LOH. The SLE patient with two unstable microsatellite markers was ∆CNV_neg_ without LOH. Thus, LDGs isolated from SLE patients displayed MSI in addition to the copy number alterations and LOH identified by cytogenetic microarray analysis, indicating that genomic alterations associated with DNA strand breaks and replication error are present in LDGs (see Additional file 5).

## Discussion

We have found evidence for multiple forms of genomic instability within an abnormal pool of neutrophils isolated from human SLE patients. Cytogenetic microarray analysis revealed genomic instability within the LDGs of SLE patients (see Additional file 6). Of the thirteen patients analyzed, six had pronounced alterations in copy number within their LDG fraction relative to autologous normal-density neutrophils. This is consistent with reports in patients with myelodysplastic syndromes, where cytogenetic microarray analysis detects an increased frequency of somatic duplications and deletions in myeloid progenitors, particularly in cases where classical cytogenetics is uninformative [53–58]. The copy number alterations and LOH are consistent with an increase in DNA strand break repair within the LDGs [59, 60]. In addition, three of the thirteen SLE patients also displayed MSI, a feature associated with replication error-prone cells [61, 62]. Thus, there was evidence for multiple types of DNA damage in the LDGs consistent with genomic instability in SLE. It is unlikely these genomic alterations occur secondary to *in situ* activation of the LDGs, resulting in NETosis and random damage of genomic DNA, which is subsequently detected as a genomic alteration. This alternative mechanism is not supported due to the presence of genomic alterations beyond deletions. The cytogenetic microarrays identified significant increases in the levels of copy number gains and LOH, and MSI was detected by an independent assay technique. The extent and types of genomic alterations that are found in the LDGs are not consistent with detection of activation-induced damage. The notion that genomic instability can be associated with SLE is supported by several observations. SLE patients are at an increased risk for certain cancers, including lymphomas and myeloid leukemia, beyond that associated with drug therapy for the disease [63, 64]. Lymphoblastoid cell lines prepared from a subset of pediatric SLE patients display an increased susceptibility to irradiation-induced double-stranded DNA breaks [21], an observation that is consistent with the alterations in copy number that are present in the LDGs.

The copy number alterations were selectively distributed on chromosomes 19, 17, 8 and X, and the copy number alterations on chromosome 19 were clustered within a few genomic intervals, consistent with a nonrandom pattern of damage. In addition to the accumulated somatic copy number alterations, these SLE patients also had an increased frequency of LOH. Several patients had an LOH that included several genes on chromosome 5q. This particular interval encodes several cytokines, the DNA repair enzyme RAD50, and IRF1. The transcription factor IRF1 contributes to the development autoimmunity in animal models [65–67], and loss of IRF1 tumor suppressor function has been proposed to promote myelodysplasias and the development of myelodysplasia-associated autoimmunity [49, 50, 68]. The region of LOH at chromosome 5q is contained within a larger interval that is associated with a distinct type myelodysplasia, 5q-syndrome [50, 68, 69]. 5q-syndrome is distinguished from other myelodysplasias in that it typically has a milder clinical course, infrequently converts to acute myeloid leukemia, is more prevalent in females, and is responsive to lenalidomide therapy [70, 71].

While previous studies have examined the functional differences between LDGs and autologous normal-density neutrophils, their developmental basis remained unexplored. We examined a model in which the LDGs arise from abnormal myeloid development in a manner resembling myelodysplasia, thus the techniques relied on the comparison of each patient’s LDGs to their autologous normal-density neutrophils. This experimental design is better suited for interpretation of data from individual SLE patients, rather than from a pooled cohort of patients, since it is differentiates genomic copy number variants from somatic alterations [72–74]. While the genomic techniques utilized in this study are suitable for evaluation of genome-wide alterations, they lack the necessary degree of fine specificity required for an associated functional analysis. For example, the consequence of a copy number loss within a heterozygous lupus susceptibility interval may depend upon whether the wild-type or the risk allele was lost. Thus, cytogenetic microarray analysis alone may not be sufficient to establish the relationship between genomic alterations and functional consequences. In addition, the incidence of microsatellite instability is consistent with the potential to accumulate point mutations that cannot be detected by the genomic microarray. Analysis of point mutations would necessitate the use of a next-generation sequencing-based screening panel, or whole exome sequencing. Although this study has focused on abnormal neutrophil development, it is certainly feasible to design related studies to examine genomic alterations in other cell lineages, including isolated subsets of abnormal T- and B-lymphocytes. This genomic analysis may provide similar insights into the role of genomic alterations in the development of autoimmunity as recently described for autoimmune lymphoproliferative syndrome, or ALPS [75–77].

The similarity between the alterations in myeloid development in myelodysplasia and SLE has been reported previously. Bone marrow biopsies from SLE patients resemble those from MDS patients diagnosed with refractory anemia, with abnormal regions of myeloid precursors and alterations in neutrophil morphology [78, 79], and the diagnostic criteria for MDS exclude SLE as a cause of observed cytopenia. Recent advances in myelodysplasia research have identified several new genomic alterations that may also be relevant to SLE. Genome-wide exome sequencing has revealed that patients with myelodysplasia frequently harbor somatic mutations in proteins that form the spliceosome complex [80, 81], and these mutations are strongly associated with the type of myelodysplasia and long-term prognosis. The possibility that spliceosome proteins may also be mutated in LDGs opens an exciting new area for future research, and a more direct and detailed analysis of specific spliceosome proteins in SLE seems warranted. In addition to promoting abnormal immune cell development, genetic alterations in the spliceosome machinery may also lead to dysregulated expression of autoantigens, and the subsequent development of autoimmunity, a feature that has been associated with myeloid leukemias.

The advances in the genetics of SLE are occurring in conjunction with research defining a key role for excessive activation of the Type 1 interferon pathway [2, 82]. Type 1 interferons participate in anti-viral immune responses, and the relationship between Type 1 interferon gene expression signature and the development of SLE is observed in several mouse models and in many SLE patients with active disease [17, 83–88]. As such, genes that regulate the expression of, or the response to, Type 1 interferons are frequently given high priority as candidate genes in genetic analysis [89–91]. While this strategy has identified variants and haplotypes of signal transducer and activator of transcription 4 (STAT4) and interferon regulatory factor 5 (IRF5) [92, 93], definitive associations within other genetic intervals, including the prominent association within the MHC locus [94–96], have yet to be established through this candidate gene selection process. Because the relative risk of disease that is associated with any one interval in isolation, it is generally interpreted as supportive evidence of the polygenetic nature of the disease. However, it is also possible that the variant that conveys the true lupus susceptibility within the interval may not have been identified. Because the screening of candidate genes within the larger susceptibility intervals generally focuses on genes associated with immune cell function or inflammatory responses, a potential selection bias may be introduced into the screening process.

Influences beyond genetics are also thought to be critical for the development of SLE. Despite the current interest in genetics, the disease concordance in identical twins is relatively low compared to other inherited diseases. This is typically attributed to a role for environmental influences in SLE, however, the exact nature of the environmental factor that drives disease development in a genetically susceptible individual remains unresolved. Our results suggest that one of the as yet undefined environmental components may be related to developmental alterations that are attributable to genetic instability or DNA damage. Recent high-resolution mapping of the MHC locus has identified a susceptibility interval that includes the DNA repair gene MSH5, consistent with a role for DNA damage and repair in the development of SLE [94, 97, 98].

We have identified multiple types of genomic alterations in LDGs isolated from human SLE patients (copy number gains and losses, copy number-neutral LOH, and MSI) and found that these alterations are clustered on certain chromosomes in areas that are potentially involved in granulocyte development and immune response regulation. A recent study has found that SLE patients have an increased risk of developing myelodysplastic disorders and myeloid leukemias [99]. One of the implications of this research is that candidate genes related to DNA damage repair should be included in the interpretation of genome-wide association studies, with affected individuals harboring the susceptibility gene likely displaying a mutator phenotype [100]. The use of cytogenetic approaches that have been instrumental in understanding the development of cancer may also be applicable to understand role of genetic instability in SLE, and to guide the development of potential new therapeutic strategies for controlling the disease and its manifestations. Although the current emphasis for new chemotherapeutic agents for the treatment of SLE is focused almost exclusively on developing antagonists of cytokines and growth factors that are associated with disease severity, this research suggests a role for the use of anti-myelodysplastic agents as well.

## Conclusions

The peripheral blood mononuclear cell fraction isolated from patients with SLE contains a pool of low-density granulocytes (LDGs). Previous studies have proposed that these abnormal neutrophils are either immature polymorphonuclear cells, or neutrophils that have been activated *in situ*. However, since LDGs express surface markers that are characteristic of a fully mature neutrophil, and have a gene expression profile that is not consistent with marked activation, an alternative mechanism likely mediates the presence of LDGs. Because LDGs resemble the abnormal neutrophils present in myelodysplasias, we applied a genomic analysis approach that is more commonly used in cancer research to determine whether LDGs display evidence of genomic instability. Numerous genomic alterations were identified in LDGs isolated from SLE patients, including copy number alterations, losses of heterozygosity, and microsatellite instability. Taken together, this supports a model whereby genomic damage contributes to the development of an abnormal population of neutrophils. Moreover, the presence of genomic instability suggests a confounding factor in the interpretation of genetic association studies. These findings also suggest that therapeutic approaches designed to control myelodysplasias may also be beneficial in SLE.

## Electronic supplementary material

Additional file 1: **Intact genomic DNA isolated from autologous pairs of SLE neutrophils and LDGs.** Genomic DNA isolated from SLE normal density neutrophils (top) and LDGs (bottom), DNA is intact and high quality (major band is >24 kb with no smearing or laddering). Yield and quality of DNA from LDGs and autologous normal density neutrophils is suitable for cytogenetic microarray analysis. (PDF 132 KB)

Additional file 2: **Cytogenetic microarray results for the SLE ∆CNV**_**neg**_ **donor SLE6.** Six copy number variations were detected in the LDG sample (red line adjacent to ideogram) as well as the autologous normal density neutrophils (blue line). These CNVs are single copy alterations which were comprised of five deletions (red downward triangles located at 2q, 5p, 14q, 17p and 21q) and one duplication (blue upward triangle located at 10q). All CNVs were present in both the SLE LDGs and the autologous neutrophil samples consistent with a pattern of inheritance rather than somatic alterations due to DNA damage. This patient was also negative for 5q LOH, MSI, JAK2 V617F somatic mutations, and activating mutations in Flt3 kinase. See Additional file 5 for clinical information. (PDF 139 KB)

Additional file 3: **JAK2 V617F somatic mutation was not present in SLE LDGs or normal density neutrophils.** JAK2 V617F somatic mutation is associated with myeloproliferative disorders, and promotes clonotypic expansion from the altered progenitor. Tetra-primer ARMS PCR primers designed to differentiate JAK2 V617F and wild-type JAK2 were used to examine LDGs and neutrophil samples from SLE patients, identical results were observed with all 13 SLE patients, and all control neutrophils. PCR products from the JAK2 V617F-positive erythroleukemic cell line HEL, and the wild-type JAK2-expressing cell line Jurkat, were included as reference standards. (PDF 1 MB)

Additional file 4: **The receptor tyrosine kinase Flt3 did not have an activating D835 mutation in LDGs or normal-density neutrophils.** Shown are LDG and neutrophil samples from two SLE patients, identical results were observed with all 13 SLE patients as well as all control neutrophils. EcoRV cut and uncut Flt3 PCR products from the T-lymphocytic cell line Jurkat were included as a reference. (PDF 63 KB)

Additional file 5: **Summary of patients, genomic alterations, and clinical characteristics.** Average age of the patients was 39.1 yr (range: 23 to 63). Average time from original diagnosis was 7.6 yr (range: 1 to 25). HCQ, hydroxychloroquine; MMF, mycophenolate; MTX, methotrexate; Pred, prednisone; Im, imuran; Qui, quinacrine; ND, not determined. (PDF 45 KB)

Additional file 6: **Copy number variations detected in SLE samples.** For each autologous pair of SLE neutrophils and LDGs, the type of CNV (copy number gain or loss), the affected chromosome (Ch), the position of the CNV start and stop, total size of CNV in kb, the number of contiguous microarray markers detecting the CNV, and the confidence are indicated. (PDF 154 KB)

## Abbreviations

ARMS: amplification refractory mutation system
CNV: copy number variation
LDG: low-density granulocyte
LOH: loss of heterozygosity
MSI: microsatellite instability
SLE: systemic lupus erythematosus.
