# Supplementary figures and images for: Genomic alterations in abnormal neutrophils isolated from adult patients with systemic lupus erythematosus
Source: Arthritis Res Ther. 2014 Aug 8;16(4):R165. doi: 10.1186/ar4681 (PMC4262380; doi:10.1186/ar4681)

## Additional File 1

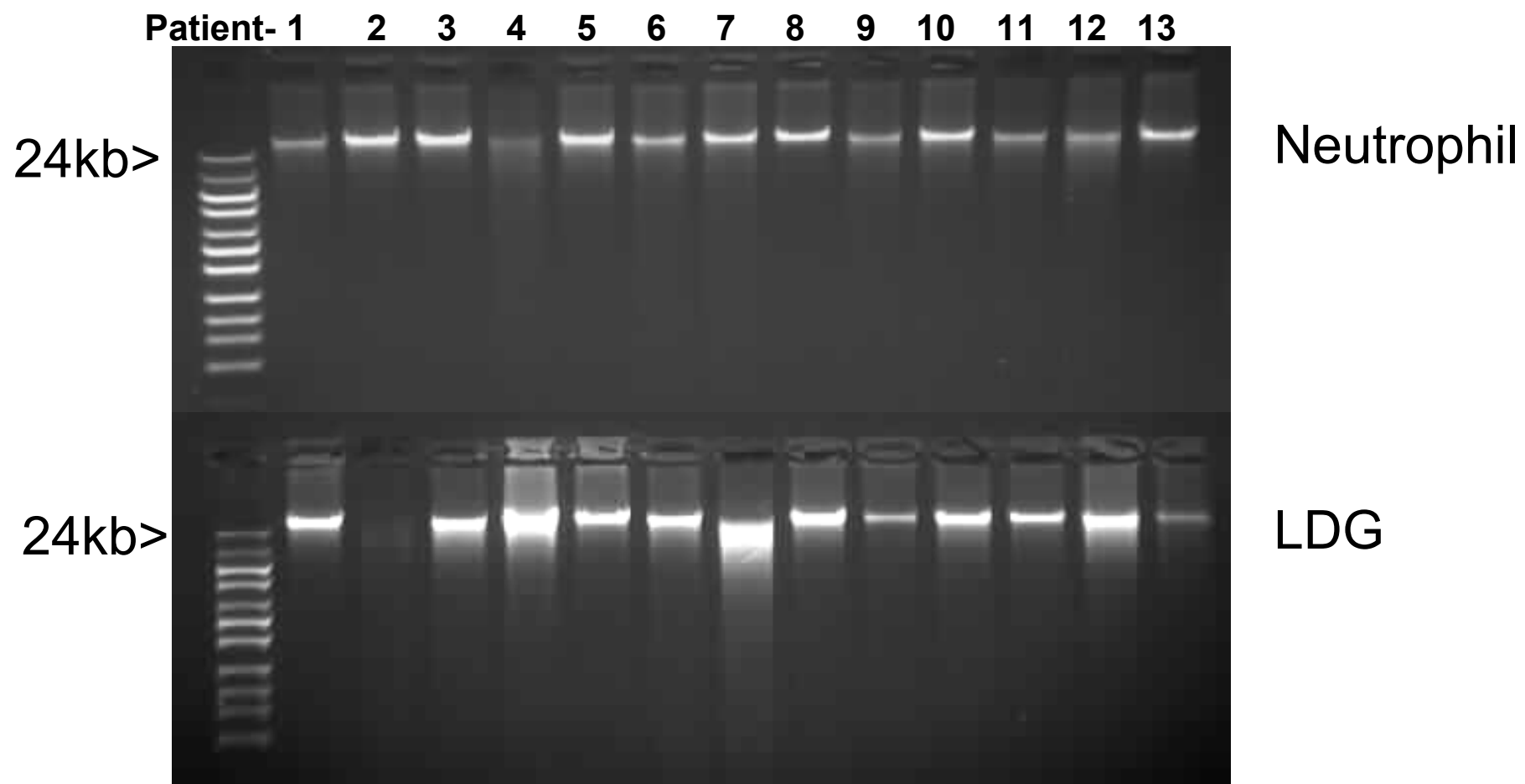

Supplement: Supplementary file 1 — Additional file 1: Intact genomic DNA isolated from autologous pairs of SLE neutrophils and LDGs. Genomic DNA isolated from SLE normal density neutrophils (top) and LDGs (bottom), DNA is intact and high quality (major band is >24 kb with no smearing or laddering). Yield and quality of DNA from LDGs and autologous normal density neutrophils is suitable for cytogenetic microarray analysis. (PDF 132 KB) [file 13075_2014_4352_MOESM1_ESM.pdf]

## Additional File 2

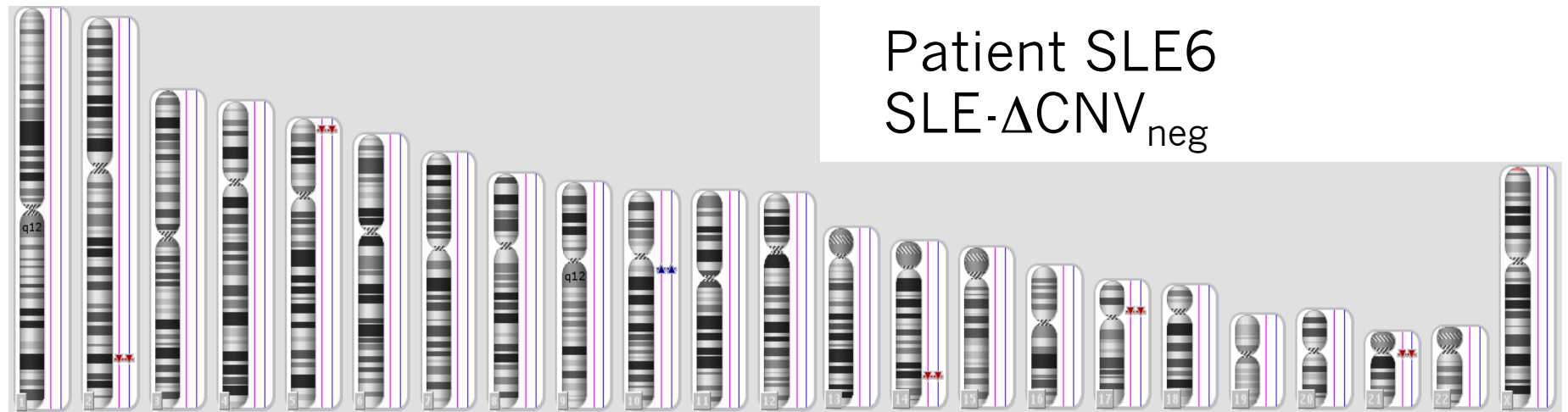

Supplement: Supplementary file 2 — Additional file 2: Cytogenetic microarray results for the SLE ∆CNV neg donor SLE6. Six copy number variations were detected in the LDG sample (red line adjacent to ideogram) as well as the autologous normal density neutrophils (blue line). These CNVs are single copy alterations which were comprised of five deletions (red downward triangles located at 2q, 5p, 14q, 17p and 21q) and one duplication (blue upward triangle located at 10q). All CNVs were present in both the SLE LDGs and the autologous neutrophil samples consistent with a pattern of inheritance rather than somatic alterations due to DNA damage. This patient was also negative for 5q LOH, MSI, JAK2 V617F somatic mutations, and activating mutations in Flt3 kinase. See Additional file 5 for clinical information. (PDF 139 KB) [file 13075_2014_4352_MOESM2_ESM.pdf]

### Additional File 3

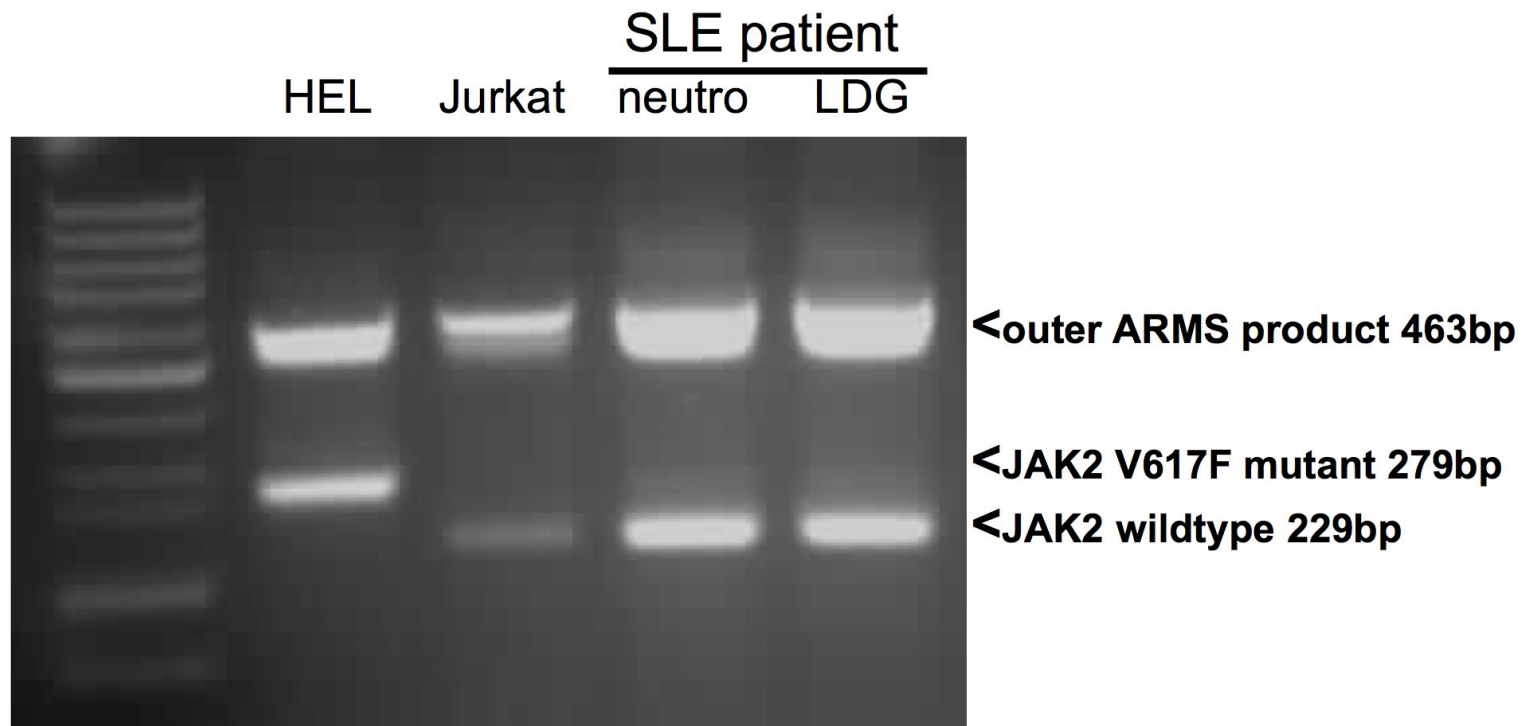

Supplement: Supplementary file 3 — Additional file 3: JAK2 V617F somatic mutation was not present in SLE LDGs or normal density neutrophils. JAK2 V617F somatic mutation is associated with myeloproliferative disorders, and promotes clonotypic expansion from the altered progenitor. Tetra-primer ARMS PCR primers designed to differentiate JAK2 V617F and wild-type JAK2 were used to examine LDGs and neutrophil samples from SLE patients, identical results were observed with all 13 SLE patients, and all control neutrophils. PCR products from the JAK2 V617F-positive erythroleukemic cell line HEL, and the wild-type JAK2-expressing cell line Jurkat, were included as reference standards. (PDF 1 MB) [file 13075_2014_4352_MOESM3_ESM.pdf]

Additional File 4

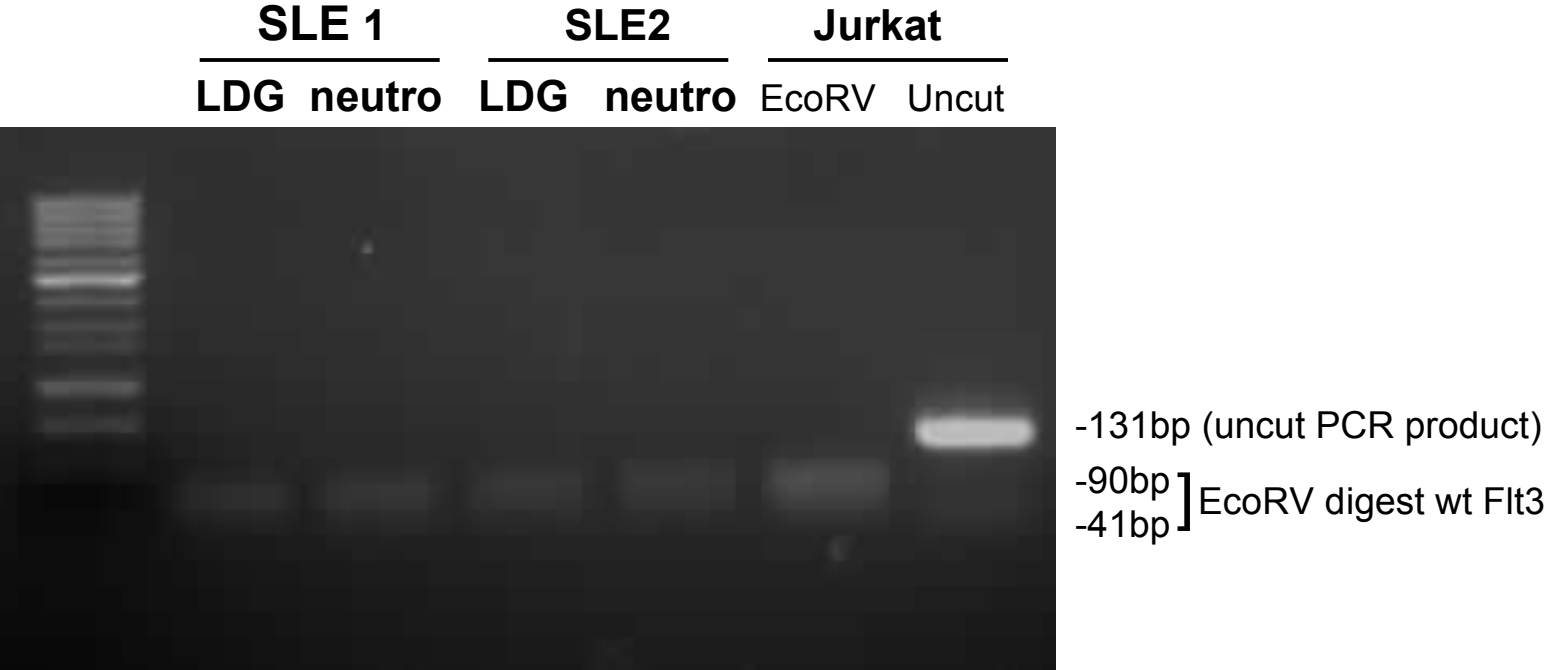

Supplement: Supplementary file 4 — Additional file 4: The receptor tyrosine kinase Flt3 did not have an activating D835 mutation in LDGs or normal-density neutrophils. Shown are LDG and neutrophil samples from two SLE patients, identical results were observed with all 13 SLE patients as well as all control neutrophils. EcoRV cut and uncut Flt3 PCR products from the T-lymphocytic cell line Jurkat were included as a reference. (PDF 63 KB) [file 13075_2014_4352_MOESM4_ESM.pdf]
