# Supplementary material for: Genomic alterations in abnormal neutrophils isolated from adult patients with systemic lupus erythematosus
Source: Arthritis Res Ther. 2014 Aug 8;16(4):R165. doi: 10.1186/ar4681 (PMC4262380; doi:10.1186/ar4681)
Supplement: Supplementary file 5 — Additional file 5: Summary of patients, genomic alterations, and clinical characteristics. Average age of the patients was 39.1 yr (range: 23 to 63). Average time from original diagnosis was 7.6 yr (range: 1 to 25). HCQ, hydroxychloroquine; MMF, mycophenolate; MTX, methotrexate; Pred, prednisone; Im, imuran; Qui, quinacrine; ND, not determined. (PDF 45 KB) [file 13075_2014_4352_MOESM5_ESM.pdf]

Additional File 5

ID	AGE	Post-Dx (yrs)	CNV>18	5q LOH	MSI	MEDs	Hx Cytoxan	Hx Cancer	Clinical	SLEDAI
SLE1	57	25	NO	**YES**	NO	HCQ, MMF	**2yr 1995, 6m 2003**	NO	Skin, joints, kidney, CNS	2
SLE2	63	6	NO	**YES**	NO	HCQ, MTX, Pred	NO	NO	Skin, joints, vasculopathy	0
SLE3	23	6	NO	**YES**	**1**	HCQ, MMF	NO	NO	Vasculitis, thrombosis	0
SLE4	41	8	NO	NO	**2**	HCQ, MMF, Pred	**6m 2005**	NO	Kidney, joints, CNS, lungs	9
SLE5	41	3	NO	NO	NO	HCQ	NO	NO	Skin	0
SLE6	52	8	NO	NO	NO	None	NO	NO	Skin, joints, CNS	2
SLE7	27	1	**YES**	NO	NO	HCQ, MMF, MTX	NO	NO	Pancytopenia, lymphad., kidney	6
SLE8	40	7	**YES**	NO	NO	HCQ, Pred	NO	NO	Joints, CNS, lymphopenia	3
SLE9	37	4	**YES**	NO	NO	HCQ	NO	NO	Skin, joints, mucositis	0
SLE10	43	10	**YES**	NO	NO	Pred	**1yr 2000**	NO	Skin, joints, lung, neutropenia	0
SLE11	27	10	**YES**	**YES**	**3**	HCQ	**6m 1998**	**YES, vulva**	Skin, joints, lung, blood	6
SLE12	27	7	**YES**	NO	NO	MMF, Pred	NO	NO	CNS, kidney, serositis	ND
SLE13	30	4	NO	NO	NO	HCQ, Pred, Im, Qui	NO	NO	CNS, kidney, thrombosis	ND
