# Supplementary material for: Genomic alterations in abnormal neutrophils isolated from adult patients with systemic lupus erythematosus
Source: Arthritis Res Ther. 2014 Aug 8;16(4):R165. doi: 10.1186/ar4681 (PMC4262380; doi:10.1186/ar4681)
Supplement: Supplementary file 6 — Additional file 6: Copy number variations detected in SLE samples. For each autologous pair of SLE neutrophils and LDGs, the type of CNV (copy number gain or loss), the affected chromosome (Ch), the position of the CNV start and stop, total size of CNV in kb, the number of contiguous microarray markers detecting the CNV, and the confidence are indicated. (PDF 154 KB) [file 13075_2014_4352_MOESM6_ESM.pdf]

Gain/Loss	Ch	CNV start (bp)	CNV stop (bp)	Size (kb)	Markers	Confidence (%)
**SLE1-LDG**						
Loss	20	14706062	15049014	343.0	461	0.88
Loss	19	43655063	43696910	41.8	25	0.88
Loss	7	111114978	111192742	77.8	87	0.89
Loss	4	28782016	28864288	82.3	60	0.90
Loss	2	154483214	154522807	39.6	35	0.92
**SLE1-neutro**						
Gain	20	30881573	30891913	10.3	15	0.94
Loss	20	14702914	15044555	341.6	461	0.89
Loss	19	43650604	43696910	46.3	28	0.90
Loss	7	111114978	111192742	77.8	87	0.88
Loss	4	28782016	28861544	79.5	58	0.89
Loss	2	154483949	154535645	51.7	38	0.91

Gain/Loss	Ch	CNV start (bp)	CNV stop (bp)	Size (kb)	Markers	Confidence (%)
**SLE2-LDG**						
Gain	X	144050207	144122679	72.5	58	0.91
Gain	15	57643002	57780056	137.1	140	0.90
Gain	6	27627956	27658827	30.9	28	0.92
Gain	5	32109590	32166970	57.4	53	0.97
Loss	X	102809170	102825846	16.7	14	0.92
Loss	X	111712583	111742229	29.6	23	0.95
Loss	19	9056289	9078634	22.3	24	0.88
Loss	19	22899584	22917823	18.2	17	0.89
Loss	19	23197661	23255507	57.8	29	0.87
Loss	19	22208351	22233538	25.2	21	0.88
Loss	15	20175623	22380045	2204.4	27	0.87
Loss	4	35375583	35412187	36.6	33	0.92
**SLE2-neutro**						
Gain	X	144050207	144122679	72.5	58	0.92
Gain	15	57643590	57778897	135.3	137	0.89
Gain	5	32109590	32168881	59.3	54	0.96
Loss	X	111712583	111751441	38.9	25	0.92
Loss	X	36767338	36808401	41.1	31	0.91
Loss	19	23488182	23501266	13.1	13	0.89
Loss	4	35375583	35412187	36.6	33	0.91

Gain/Loss	Ch	CNV start (bp)	CNV stop (bp)	Size (kb)	Markers	Confidence (%)
**SLE3-LDG**						
Gain	16	77201276	77220779	19.5	16	0.94
Gain	15	43874345	44014976	140.6	78	0.88
Gain	8	73595715	73637008	41.3	49	0.89
Gain	4	58052306	58098367	46.1	34	0.92

Loss	21	24020827	24043278	22.5	27	0.94
Loss	20	59874661	59894458	19.8	16	0.87
Loss	19	54181614	54209600	28.0	31	0.86
Loss	19	22900427	22917823	17.4	16	0.90
Loss	4	134921202	135187273	266.1	181	0.92
Loss	1	102656252	102858574	202.3	193	0.94
**SLE3-neutro**						
Loss	X	70918973	70951706	32.7	22	0.87
Loss	21	24020827	24043278	22.5	27	0.91
Loss	4	134926438	135187273	260.8	177	0.89
Loss	1	102656252	102854997	198.7	190	0.90

Gain/Loss	*Ch*	*CNV start (bp)*	*CNV stop (bp)*	*Size (kb)*	*Markers*	*Confidence (%)*
**SLE4-LDG**						
Gain	17	46610961	46713470	102.5	75	0.87
Gain	15	32028803	32439298	410.5	232	0.89
Gain	8	113956886	113985846	29.0	26	0.90
Loss	19	43351508	43385662	34.2	18	0.88
Loss	19	22899584	22922772	23.2	22	0.87
Loss	19	23195946	23210451	14.5	13	0.89
Loss	14	86288075	86311694	23.6	16	0.90
Loss	12	7991289	8124521	133.2	35	0.88
Loss	10	47082046	47140933	58.9	29	0.87
Loss	8	137682483	137850448	168.0	56	0.89
Loss	5	45858647	45881237	22.6	15	0.93
**SLE4-neutro**						
Gain	17	36853487	36866669	13.2	11	0.93
Gain	15	32028803	32427799	399.0	231	0.88
Loss	X	70918042	71005018	87.0	65	0.88
Loss	X	49188141	49370135	182.0	21	0.87
Loss	X	51422847	51462146	39.3	37	0.85
Loss	19	43372382	43421285	48.9	35	0.87
Loss	14	86288614	86311694	23.1	15	0.92
Loss	12	8001775	8124521	122.7	32	0.89
Loss	10	89144719	89159812	15.1	10	0.91
Loss	10	49201540	49377892	176.4	15	0.89
Loss	8	137682483	137850448	168.0	56	0.89
Loss	5	45861783	45881237	19.5	12	0.93

Gain/Loss	*Ch*	*CNV start (bp)*	*CNV stop (bp)*	*Size (kb)*	*Markers*	*Confidence (%)*
**SLE5-LDG**						
Gain	7	14883488	15608591	725.1	791	0.88
Gain	2	129025909	129051292	25.4	24	0.91
Loss	19	43410504	43431575	21.1	16	0.88
Loss	15	20175623	22330871	2155.2	23	0.87

Gain/Loss	Ch	CNV start (bp)	CNV stop (bp)	Size (kb)	Markers	Confidence (%)
Loss	8	8525854	8546141	20.3	24	0.89
**SLE5-neutro**						
Gain	7	14883488	15611978	728.5	796	0.89
Gain	6	56791600	56807330	15.7	19	0.93
Loss	X	36771111	36807911	36.8	27	0.91
Loss	X	105606252	105637564	31.3	26	0.88
Loss	X	17782435	17794539	12.1	10	0.93
Loss	X	3350787	3366713	15.9	17	0.88
Loss	15	20175623	22483277	2307.7	44	0.87
Loss	8	8518847	8543985	25.1	28	0.89
Loss	2	74591677	74604271	12.6	17	0.89

Gain/Loss	*Ch*	*CNV start (bp)*	*CNV stop (bp)*	*Size (kb)*	*Markers*	*Confidence (%)*
**SLE6-LDG**						
Gain	X	64911279	64923873	12.6	22	0.93
Gain	X	48569318	48587694	18.4	20	0.93
Gain	22	23024245	23207076	182.8	11	0.94
Gain	18	60815399	60832599	17.2	34	0.91
Gain	17	38465881	38511158	45.3	37	0.89
Gain	17	56393125	56417479	24.4	16	0.93
Gain	17	42509379	42544758	35.4	40	0.89
Gain	17	17396528	17450723	54.2	31	0.90
Gain	16	21398654	21952747	554.1	121	0.89
Gain	16	75129262	75154351	25.1	10	0.96
Gain	15	40459241	40492513	33.3	45	0.89
Gain	8	110527817	110555748	27.9	28	0.89
Gain	8	107536759	107556752	20.0	19	0.92
Gain	7	39694883	39712377	17.5	25	0.90
Gain	6	41670328	41680717	10.4	34	0.91
Gain	3	193373964	193407138	33.2	37	0.89
Gain	2	183991948	184022278	30.3	41	0.88
Gain	1	41829858	41855780	25.9	17	0.92
Gain	1	25668981	25682555	13.6	16	0.93
Gain	1	155940222	155963400	23.2	17	0.93
Gain	1	156710845	156720205	9.4	15	0.93
Loss	X	49168695	49370135	201.4	24	0.94
Loss	X	51422847	51462783	39.9	38	0.90
Loss	X	13329658	13368071	38.4	41	0.88
Loss	X	62380380	62436535	56.2	34	0.87
Loss	X	151556909	151566122	9.2	21	0.91
Loss	X	70918042	71016435	98.4	71	0.89
Loss	X	64575482	64651335	75.9	22	0.90
Loss	22	26713042	26733231	20.2	30	0.86
Loss	21	22626491	22641192	14.7	21	0.88
Loss	21	46500645	46542670	42.0	21	0.87
Loss	20	59877405	59894458	17.1	12	0.89

Gain/Loss	Ch	CNV start (bp)	CNV stop (bp)	Size (kb)	Markers	Confidence (%)
Loss	20	6291314	6306581	15.3	11	0.91
Loss	19	38167363	38178285	10.9	14	0.89
Loss	15	44639774	44658052	18.3	14	0.89
Loss	11	94100120	94135355	35.2	19	0.88
Loss	10	2393383	2413720	20.3	17	0.88
Loss	8	814528	1617956	803.4	140	0.87
Loss	8	9721405	9747719	26.3	15	0.91
Loss	8	145871983	145948276	76.3	18	0.88
Loss	8	117418374	117430215	11.8	11	0.89
Loss	7	11634200	11648705	14.5	16	0.89
Loss	6	163476463	163489155	12.7	17	0.91
Loss	6	73688303	73708051	19.7	17	0.89
Loss	6	168414542	168558211	143.7	27	0.88
Loss	5	67031976	67065114	33.1	18	0.89
Loss	4	187212530	187247272	34.7	33	0.87
Loss	3	58822387	58844536	22.1	17	0.89
Loss	2	52790492	52825392	34.9	27	0.86
Loss	2	238975281	239091902	116.6	24	0.87
Loss	1	247156351	247167077	10.7	14	0.92
Loss	1	228218194	228406894	188.7	22	0.91
**SLE6-neutro**						
Gain	16	8714024	8764691	50.7	17	0.94
Gain	16	21527622	21737980	210.4	98	0.91
Gain	1	25668981	25688288	19.3	21	0.92
Loss	X	36767338	36808401	41.1	31	0.86
Loss	19	22212859	22232901	20.0	18	0.88
Loss	19	23190705	23247789	57.1	30	0.88
Loss	19	57120355	57134453	14.1	26	0.88
Loss	19	9058102	9090149	32.0	35	0.87
Loss	19	22899584	22921155	21.6	20	0.89
Loss	6	163476463	163489429	13.0	18	0.92
Loss	5	27609773	27629227	19.5	11	0.93
Loss	3	164098415	164111756	13.3	11	0.93
Loss	2	216601495	216612129	10.6	11	0.93
Loss	1	148954194	149159064	204.9	17	0.88
Loss	1	22307630	22342274	34.6	22	0.89
**SLE8-LDG**						
Gain	22	25748673	25915225	166.6	34	0.89
Gain	20	57456364	57472682	16.3	33	0.90
Gain	7	112444054	112478257	34.2	30	0.91
Gain	3	100335120	100445567	110.4	99	0.91
Loss	X	151556321	151566122	9.8	23	0.88
Loss	22	35999919	36010896	11.0	17	0.88
Loss	19	22208351	22233538	25.2	21	0.90

Loss	19	23195946	23249651	53.7	29	0.89
Loss	19	22900427	22922772	22.3	21	0.89
Loss	19	9058102	9086318	28.2	28	0.88
Loss	19	40072526	40100130	27.6	39	0.86
Loss	19	23487005	23500776	13.8	13	0.91
Loss	17	19498957	19532866	33.9	26	0.88
Loss	12	100934844	100944816	10.0	11	0.93
Loss	7	126800319	126809973	9.7	14	0.89
Loss	6	162744795	162815503	70.7	79	0.89
Loss	5	113320084	113335880	15.8	18	0.87
Loss	1	239487263	239537342	50.1	58	0.95
**SLE8-neutro**						
Gain	22	25671645	25822762	151.1	28	0.90
Gain	7	112449445	112478061	28.6	27	0.91
Gain	3	100335120	100446645	111.5	101	0.91
Loss	19	23197661	23247789	50.1	27	0.87
Loss	19	22896703	22920224	23.5	21	0.87
Loss	18	64169645	64180181	10.5	10	0.94
Loss	17	19498957	19535463	36.5	28	0.89
Loss	13	53161289	53176823	15.5	14	0.89
Loss	12	100934844	100943885	9.0	10	0.94
Loss	6	162745251	162815503	70.3	78	0.90
Loss	1	239487263	239535823	48.6	57	0.94

Gain/Loss	*Ch*	*CNV start (bp)*	*CNV stop (bp)*	*Size (kb)*	*Markers*	*Confidence (%)*
**SLE9-LDG**						
Gain	X	115564247	115581300	17.1	24	0.91
Gain	22	22731757	23252271	520.5	43	0.89
Gain	20	57457589	57483364	25.8	61	0.89
Gain	18	56328861	56345571	16.7	22	0.91
Gain	17	9126357	9146497	20.1	19	0.92
Gain	10	43730340	43768267	37.9	32	0.90
Gain	7	116134440	116166487	32.0	30	0.90
Gain	5	173299666	173350405	50.7	45	0.89
Gain	3	63841604	63908637	67.0	64	0.88
Gain	1	25221023	25284234	63.2	19	0.92
Gain	1	112129887	112174679	44.8	39	0.89
Loss	X	49168695	49355288	186.6	23	0.91
Loss	X	47887189	47991658	104.5	79	0.88
Loss	X	36743867	36807127	63.3	43	0.90
Loss	X	108462041	108507053	45.0	29	0.87
Loss	X	16339434	16356291	16.9	13	0.93
Loss	X	3771831	3818199	46.4	11	0.91
Loss	X	6468412	8119292	1650.9	1143	0.89
Loss	19	40073310	40117497	44.2	50	0.86
Loss	19	54180225	54256974	76.7	70	0.86

Loss	19	9058102	9091668	33.6	37	0.87
Loss	17	18419305	18471692	52.4	12	0.90
Loss	14	67076596	67090856	14.3	26	0.86
Loss	8	47516503	47565406	48.9	23	0.87
Loss	8	71658044	71676770	18.7	14	0.88
Loss	1	102518660	102540858	22.2	18	0.89
Loss	1	236347637	236358150	10.5	10	0.91
**SLE9-neutro**						
Loss	X	6446509	8125368	1678.9	1147	0.91
Loss	X	47880525	47991658	111.1	82	0.89
Loss	13	51172771	51182817	10.0	10	0.93
Loss	3	75446326	75615328	169.0	37	0.85

Gain/Loss	*Ch*	*CNV start (bp)*	*CNV stop (bp)*	*Size (kb)*	*Markers*	*Confidence (%)*
**SLE10-LDG**						
Gain	X	48569318	48587694	18.4	20	0.92
Gain	X	6469300	8133894	1664.6	1151	0.91
Gain	20	57459353	57472829	13.5	24	0.92
Gain	17	38464460	38520664	56.2	45	0.89
Gain	17	7472951	7485398	12.4	17	0.93
Gain	17	74250005	74274165	24.2	16	0.92
Gain	17	17624574	17646086	21.5	25	0.91
Gain	17	46620908	46701122	80.2	56	0.88
Gain	16	34476235	34751469	275.2	76	0.92
Gain	15	82450267	82478792	28.5	43	0.89
Gain	10	100012076	100040350	28.3	24	0.91
Gain	7	111723019	111746540	23.5	42	0.90
Gain	6	49411857	49435084	23.2	34	0.90
Gain	5	142768493	142796228	27.7	25	0.91
Gain	2	197021928	197034424	12.5	18	0.93
Gain	1	110733632	110775528	41.9	34	0.90
Loss	X	56083760	56108397	24.6	12	0.90
Loss	X	75727713	75838895	111.2	35	0.87
Loss	19	9075448	9091668	16.2	25	0.87
Loss	17	47206752	47224148	17.4	22	0.89
Loss	15	49839997	49852787	12.8	11	0.90
Loss	11	63110301	63165525	55.2	41	0.87
Loss	11	106956232	106983325	27.1	15	0.91
Loss	10	576219	681864	105.6	18	0.88
Loss	9	28206486	28347607	141.1	100	0.91
Loss	5	101465923	101521637	55.7	25	0.93
Loss	1	148954194	149342912	388.7	50	0.88
**SLE10-neutro**						
Gain	X	48569906	48587694	17.8	19	0.93
Gain	X	6469300	8098467	1629.2	1136	0.90

Gain/Loss	Ch	CNV start (bp)	CNV stop (bp)	Size (kb)	Markers	Confidence (%)
Gain	20	25677456	25705515	28.1	11	0.94
Gain	18	60814811	60832011	17.2	35	0.89
Gain	16	74068167	74080320	12.2	19	0.91
Gain	16	34476235	34751469	275.2	76	0.91
Gain	12	58119421	58157593	38.2	29	0.90
Loss	X	151556909	151566122	9.2	21	0.87
Loss	20	59877405	59894458	17.1	12	0.90
Loss	17	47207634	47224148	16.5	21	0.90
Loss	9	28206486	28345696	139.2	99	0.91
Loss	5	101473665	101521637	48.0	24	0.94
Loss	1	148920678	149348106	427.4	53	0.88

Gain/Loss	*Ch*	*CNV start (bp)*	*CNV stop (bp)*	*Size (kb)*	*Markers*	*Confidence (%)*
**SLE11-LDG**						
Loss	X	110342445	110355823	13.4	28	0.86
Loss	X	36740339	36808401	68.1	48	0.88
Loss	X	9360802	9402553	41.8	15	0.89
Loss	X	70918042	70993846	75.8	59	0.86
Loss	X	3771831	3818199	46.4	11	0.89
Loss	X	51444799	51461558	16.8	17	0.88
Loss	22	36000801	36012219	11.4	18	0.89
Loss	20	46513525	46543318	29.8	17	0.88
Loss	19	43379144	43436916	57.8	38	0.86
Loss	19	43256448	43281096	24.6	14	0.88
Loss	19	23195946	23210451	14.5	13	0.89
Loss	19	22176550	22233538	57.0	42	0.86
Loss	19	23488182	23502785	14.6	14	0.88
Loss	19	22897830	22919489	21.7	19	0.89
Loss	12	63927930	64110358	182.4	40	0.87
Loss	12	28747346	28763566	16.2	12	0.94
Loss	12	13527881	13562002	34.1	33	0.86
Loss	11	39874470	39896962	22.5	14	0.90
Loss	9	93600093	93612959	12.9	12	0.90
Loss	8	47528557	47563250	34.7	16	0.90
Loss	8	1378982	1562390	183.4	46	0.86
Loss	5	7163107	7193782	30.7	26	0.89
Loss	4	82793517	82809250	15.7	19	0.90
Loss	2	96077517	96103145	25.6	22	0.87
**SLE11-neutro**						
Gain	22	25811687	25975985	164.3	100	0.90
Gain	7	12513951	12563927	50.0	23	0.97
Gain	3	173239374	173302977	63.6	63	0.91
Loss	X	48569906	48588674	18.8	20	0.88
Loss	X	36747297	36796347	49.1	35	0.91
Loss	19	22897830	22920224	22.4	20	0.89
Loss	14	62654493	62665000	10.5	10	0.94

Loss	9	9904480	10010533	106.1	86	0.93
Loss	6	68233476	68453830	220.4	139	0.93
Loss	6	121513654	121525760	12.1	11	0.91
Loss	4	70242607	70277937	35.3	20	0.88
Loss	1	106165117	106215294	50.2	43	0.95

Gain/Loss	*Ch*	*CNV start (bp)*	*CNV stop (bp)*	*Size (kb)*	*Markers*	*Confidence (%)*
**SLE12-LDG**						
Gain	X	2527734	2551745	24.0	29	0.88
Gain	X	24156208	24187765	31.6	24	0.91
Gain	20	57458177	57472682	14.5	26	0.91
Gain	18	56328861	56345571	16.7	22	0.92
Gain	17	38467253	38516989	49.7	38	0.90
Gain	17	42249238	42351551	102.3	35	0.89
Gain	17	43475696	43518327	42.6	18	0.92
Gain	12	58121724	58157593	35.9	27	0.92
Gain	10	119033925	119098263	64.3	66	0.88
Loss	X	51416232	51457883	41.7	37	0.85
Loss	X	105606252	105637564	31.3	26	0.87
Loss	X	139790949	139808100	17.2	13	0.90
Loss	X	2363339	2397689	34.4	41	0.88
Loss	X	55477367	55496331	19.0	14	0.91
Loss	X	70943865	71016435	72.6	52	0.85
Loss	X	2552577	2564730	12.2	18	0.87
Loss	22	35992961	36008299	15.3	18	0.89
Loss	22	48358687	48371036	12.3	23	0.88
Loss	20	53410422	53423408	13.0	18	0.89
Loss	20	59874661	59896320	21.7	18	0.88
Loss	20	60014311	60042732	28.4	30	0.87
Loss	19	22900427	22910510	10.1	12	0.90
Loss	19	55237319	55357125	119.8	43	0.89
Loss	19	8831428	8842111	10.7	15	0.89
Loss	19	43215680	43436916	221.2	120	0.87
Loss	16	26918161	26929971	11.8	11	0.90
Loss	11	123909350	123921479	12.1	10	0.93
Loss	10	491547	629875	138.3	16	0.89
Loss	10	89134723	89160939	26.2	14	0.90
Loss	10	133291746	133314624	22.9	19	0.89
Loss	8	5456450	5465533	9.1	20	0.87
Loss	8	3918161	3938742	20.6	30	0.92
Loss	8	1465369	1553619	88.3	39	0.88
Loss	8	2103888	2226291	122.4	38	0.87
Loss	1	145021347	145063096	41.7	33	0.86
**SLE12-neutro**						
Loss	X	58127630	58259494	131.9	43	0.86
Loss	19	55246727	55351294	104.6	33	0.87

Loss	19	43406633	43446569	39.9	29	0.88
Loss	8	3918161	3938742	20.6	30	0.88

Gain/Loss	*Ch*	*CNV start (bp)*	*CNV stop (bp)*	*Size (kb)*	*Markers*	*Confidence (%)*
**SLE13-LDG**						
Gain	X	120068091	120116091	48.0	17	0.99
Gain	X	62441238	62499745	58.5	40	0.92
Gain	4	165865584	165884401	18.8	19	0.94
Loss	X	3771831	3818199	46.4	11	0.92
Loss	19	54179654	54209600	29.9	34	0.86
Loss	19	9045901	9090149	44.2	52	0.86
Loss	19	22204627	22233538	28.9	22	0.88
Loss	8	56379256	56396012	16.8	20	0.95
Loss	8	13615236	13649145	33.9	13	0.91
Loss	4	12372161	12383138	11.0	11	0.94
Loss	3	165265261	165290007	24.7	17	0.94
Loss	1	148932144	149342912	410.8	51	0.86
**SLE13-neutro**						
Gain	X	120006207	120116091	109.9	19	0.97
Gain	X	62454125	62505086	51.0	36	0.94
Gain	4	165865584	165880726	15.1	18	0.95
Loss	19	54193031	54209600	16.6	20	0.88
Loss	8	13615236	13649145	33.9	13	0.92
Loss	8	56372467	56388714	16.2	20	0.95
Loss	4	12372161	12383138	11.0	11	0.96
Loss	3	165265261	165290007	24.7	17	0.92
Loss	1	148954194	149186569	232.4	20	0.90
